# Morpho-electric diversity of human hippocampal *CA1* pyramidal neurons

**DOI:** 10.1016/j.celrep.2024.114100

**Published:** 2024-04-11

**Authors:** Eline J. Mertens, Yoni Leibner, Jean Pie, Anna A. Galakhova, Femke Waleboer, Julia Meijer, Tim S. Heistek, René Wilbers, Djai Heyer, Natalia A. Goriounova, Sander Idema, Matthijs B. Verhoog, Brian E. Kalmbach, Brian R. Lee, Ryder P. Gwinn, Ed S. Lein, Eleonora Aronica, Jonathan Ting, Huibert D. Mansvelder, Idan Segev, Christiaan P.J. de Kock

**Affiliations:** 1 Center for Neurogenomics and Cognitive Research, Vrije Universiteit Amsterdam, 1081 HV Amsterdam, the Netherlands; 2 The Edmond and Lily Safra Center for Brain Sciences, The Hebrew University of Jerusalem, Jerusalem 91904, Israel; 3 Amsterdam UMC, location VUmc, Amsterdam 1081 HV, the Netherlands; 4 Allen Institute for Brain Science, Seattle, WA 98109, USA; 5 Department of (Neuro)Pathology, Amsterdam Neuroscience, Amsterdam University Medical Centers, University of Amsterdam, 1105 AZ Amsterdam, the Netherlands; 6 Epilepsy Surgery and Functional Neurosurgery, Swedish Neuroscience Institute, Seattle, WA 98122, USA; 7 Present address: Neuroscience Institute, University of Cape Town, Cape Town 7935, South Africa; 8 Present address: Eastside Neuroscience Institute Epilepsy Center, Kirkland, WA 98034, USA; 9 These authors contributed equally; 10 These authors contributed equally; 11 Lead contact

## Abstract

Hippocampal pyramidal neuron activity underlies episodic memory and spatial navigation. Although extensively studied in rodents, extremely little is known about human hippocampal pyramidal neurons, even though the human hippocampus underwent strong evolutionary reorganization and shows lower theta rhythm frequencies. To test whether biophysical properties of human *Cornu Amonis subfield 1 (CA1)* pyramidal neurons can explain observed rhythms, we map the morpho-electric properties of individual *CA1* pyramidal neurons in human, non-pathological hippocampal slices from neurosurgery. Human *CA1* pyramidal neurons have much larger dendritic trees than mouse *CA1* pyramidal neurons, have a large number of oblique dendrites, and resonate at 2.9 Hz, optimally tuned to human theta frequencies. Morphological and biophysical properties suggest cellular diversity along a multidimensional gradient rather than discrete clustering. Across the population, dendritic architecture and a large number of oblique dendrites consistently boost memory capacity in human *CA1* pyramidal neurons by an order of magnitude compared to mouse CA1 pyramidal neurons.

## In brief

The morphological and functional properties of pyramidal neurons from human hippocampus remain largely unknown. Mertens et al. generate a multimodal dataset from human surgical tissue and find large cellular diversity in *CA1*, consistently greater size and complexity of dendritic trees relative to mouse, reliable resonance properties, and higher memory capacity.

## Graphical Abstract

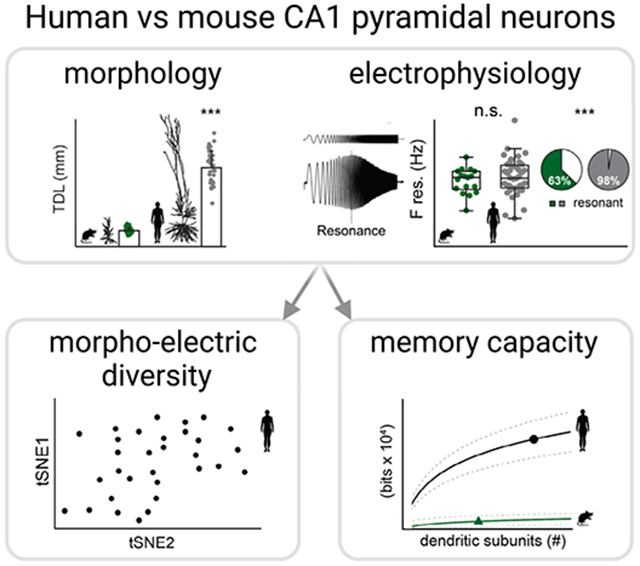

## INTRODUCTION

Navigating through an environment and remembering the steps and events along the way rely on hippocampal function.^1,2^ Although the hippocampal complex and many of its basic functions are conserved across mammals,^3,4^ the human hippocampal complex in general and *CA1* cytoarchitecture specifically show a dramatic reorganization during evolution. The increase in size and altered subregional organization in the human hippocampus are the largest in primate evolution.^5,6^ These evolutionary changes are likely associated with the emergence of specialized human cognitive abilities, such as extraordinary cognitive flexibility.^7^ Not surprisingly, human cognitive decline is consistently linked to decreased function of the hippocampal complex.^8,9^

Functionally, during spatial navigation and mnemonic processing, hippocampal networks generate a prominent theta rhythm,^10,11^ and phase locking of single neurons to the theta rhythm is associated with human memory strength and spatial navigation capabilities.^12,13^ Impaired hippocampal theta rhythm directly affects spatial navigation and cognitive performance.^14,15^ Interestingly, the theta rhythm is the only oscillatory brain rhythm that inversely scales with brain size across mammals, in contrast to alpha, beta, and gamma rhythms, which are similar across mammalian brains.^16,17^ In human hippocampus, theta rhythm is 1–4 Hz^16-19^ compared to 4–10 Hz in rats.^2,20^ A slower theta rhythm directly impacts theories on human brain function, since the slower cycle could allow an increased number of neuronal assemblies to interact and lock to the same cycle.^16,21^ This could translate to the association of an increased number of items in working memory compared to rodents and thus increased human cognitive flexibility.^22,23^

The ability of single neurons to respond with high selectivity to inputs at preferred frequencies, called “resonance,” is closely associated with the ability of brain regions to oscillate at preferred frequencies.^24,25^ Resonance is generally the outcome of a combination of passive membrane properties of the cell (capacitance and leak conductance) and voltage-gated membrane currents, including the hyperpolarization-activated current (I_h_).^26^ In rat hippocampus, pyramidal neurons in *CA1* (r*CA1*) show resonance frequency (F res) at 3–5 Hz,^26-28^ matching theta wave frequencies in rat (4–10 Hz).^2,20^ In these r*CA1* neurons, it was suggested that resonance could optimize input/output computations from synaptic pathways impinging on the apical dendrites in *CA1*, where I_h_ is particularly prominent.^29^ In addition, r*CA1* pyramidal neurons show a large number of thin, elongated oblique dendrites,^29-31^ and these oblique dendrites possess specialized computational properties.^32-35^ In view of the tight interplay between theta rhythm, resonance frequency, and cellular structure/function, it is crucial to determine how concepts and theories based on rodents translate to human hippocampus.

The subcellular structure, biophysical properties, and computational capacity of *CA1* pyramidal neurons are well documented for rodents,^20,36,37^ but these are unknown for human hippocampus. To address this and to test whether reduced theta frequencies in human hippocampus can be explained by cellular function of *CA1* pyramidal neurons, we recorded from *CA1* pyramidal neurons in acute living brain slices of non-pathological human hippocampus obtained during resection surgery.

## RESULTS

### h*CA1* pyramidal neurons have large dendritic trees with numerous oblique dendrites

**Figure 1.**
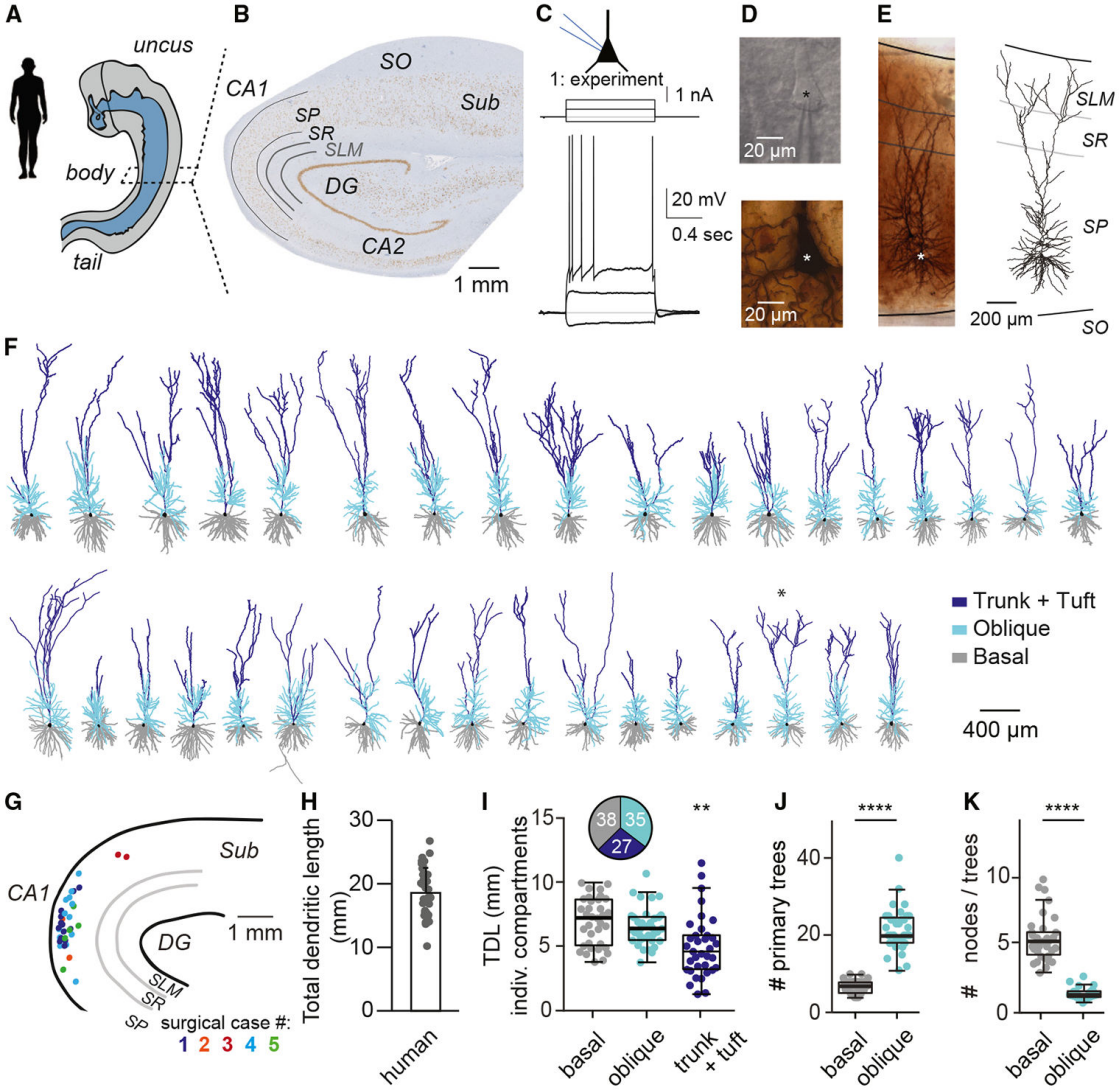
Human *CA1* pyramidal neurons have extensive dendritic trees with many oblique dendrites (A) Human hippocampal tissue originated from comparable parts of the hippocampal body. (B) Cross-section of hippocampus after NeuN histology (see STAR Methods). *SO, stratum oriens; SR, stratum radiatum; SLM, stratum lacunosum-moleculare; Sub, subiculum; CA1* and *CA2*, *Cornu Amonis subfields 1* and *2*. (C) Experimental current step protocol to quantify passive and active membrane properties. (D, top) Differential interference contrast (DIC) image of an example pyramidal cell body in *SP* during patch-clamp electrophysiology (same cell as in C). (D, bottom) Post hoc biocytin-DAB-stained cell body (same cell). (E, left) Analogous to (D) (bottom) but complete pyramidal neuron is illustrated using a collapsed z stack image with layer borders superimposed. Two pyramidal neurons are visible; the example from (C) and (D) is indicated by the asterisk. (E, right) Digital reconstruction of the example cell in (C), (D), and (E). (F) Gallery of dendritic reconstructions of human *CA1* (h*CA1*) pyramidal neurons with basal dendrites in gray, oblique dendrites in cyan, main apical trunk and tuft in blue, and soma in black. (G) Annotation of reconstructed morphologies relative to anatomical landmarks and layer position. Colors refer to surgical cases. (H) Total dendritic length (TDL) of h*CA1* pyramidal neurons. Histogram denotes mean + standard deviation. (I) Compartment-specific TDL. Pie chart inset represents the fraction (in %) of individual compartments relative to TDL of full morphology (*n* = 35, Kruskal-Wallis with Dunn’s post hoc test, *p* < 0.01). (J) Number of primary trees is significantly different for basal vs. oblique dendrites (basal: 7.0 primary trees, 5.5–8.0 and obliques: 20.0 trees, 18.5–24.5 [median, 1^st^–3^rd^ quartile range], *n* = 35, Mann-Whitney, *p* < 0.0001). (K) Number of nodes per individual tree is significantly different for basal vs. oblique dendrites (basal: 5.0 nodes/tree, 4.1–5.7 and obliques: 0.9 nodes/tree, 0.7–1.2, [median, 1^st^–3^rd^ quartile range] *n* = 35, Mann-Whitney, *p* < 0.0001). Scale bar: 400 μm. See also Figure S1.

Tissue samples were evaluated for structural abnormalities, and only non-sclerotic samples without evident structural alterations (and that were not part of deep brain pathology) were included in this study (*n* = 6 cases). We used small tissue blocks (±1–2 cm^3^) from human hippocampal bodies for cytoarchitectural assessment, electrophysiological recordings, and post hoc histology (Figure 1). These tissue blocks were obtained during en bloc resection of mesio-temporal structures for surgical treatment of drug-resistant epilepsy and, in one case, to remove a deep brain tumor. NeuN-DAB (see STAR Methods) staining revealed numerous cell bodies throughout the *stratum pyramidale (SP)* across canonical regions of the hippocampus (Figure 1B). The homogeneous distribution of neuronal somata in human *CA1* (h*CA1*) of the tissue samples confirmed that the tissue was non-sclerotic. All resected samples were evaluated by an expert pathologist, and only samples without evident structural alterations were included (Table S1). The thickness of the human *SP* was 1,111 μm (median; 993–1,232 μm, 1^st^–3^rd^ quartile range; *n* = 6 surgical cases), which not only exceeds the mouse *SP* thickness by a factor of 15 (mouse *SP*: 75 μm^38^) but also exceeds the *SP* thickness of rat (5 cell bodies thick) and macaque (10–15 cell bodies thick).^32^

Tissue originated from the hippocampal body, through visual inspection by a neurosurgeon upon resection and anatomical origin, was comparable among subjects. We made stable whole-cell patch-clamp recordings of pyramidal neurons (*n* = 41 recordings, recording duration 30–60 min, Figure 1C) and probed passive and active membrane properties using a variety of stimulus protocols. Differential interference contrast microscopy indicated the presence of healthy somata, based on their appearance (Figure 1D, top) with an average diameter of 19 ± 3 μm and an average surface area of 330 ± 60 μm^2^(*n* = 32), matching previously reported soma size from healthy subjects.^38^ Recorded neurons were dye filled with biocytin for post hoc histology (Figure 1D, bottom) and digital reconstruction (Figure 1E, right). Additionally, cytoarchitectural layers were determined to annotate reconstructed neurons to anatomical landmarks (Figures 1E, right, and S1; see STAR Methods). Thus, hippocampal slices contained healthy neurons with stable, intact membranes, and neurons showed repetitive action potential (AP) spiking in response to current injection. We did not observe spontaneous epileptiform activity in slices.

The tissue samples used for patch-clamp electrophysiology contained intact *CA1, CA2*, and *CA3* regions in addition to the *dentate gyrus (DG)* and at least part of the *subiculum* (Figure 1B). Within the region of interest of the current study (i.e., *CA1*), samples always included all canonical, cytoarchitectural layers of hippocampus *CA1* (Figures 1B, 1E, and S1). Anatomical landmarks (*DG*) and layer borders subsequently allowed the annotation of *n* = 35 individual reconstructed neurons to a standardized framework, including cellular position within the *SP* with respect to the *DG* apex and radial position in *SP* relative to *stratum oriens* and *stratum lacunosum-moleculare (SLM)* borders (Figures 1F, 1G, S1A, and S1B).

The reconstructed h*CA1* pyramidal neurons showed extensive dendritic trees that were relatively intact (Figures 1F and S1). The total dendritic length (TDL) of single h*CA1* pyramidal neurons accumulated to 18.6 ± 3.9 mm (average ± SD, *n* = 35, Figure 1H). This significantly exceeds the TDL in rat (12.9 ± 1.5 mm, *n* = 29),^39,40^ hamster (3.0 ± 0.6 mm, *n* = 66),^41^ or mouse (3.7 ± 0.6 mm, *n* = 46,^38^ ANOVA, *p* < 0.001; Figure S1C). Thus, h*CA1* pyramidal neurons have extended dendrites compared to rodent species.

Next, we quantified the length of basal, oblique, and apical trunk + tuft dendrites of h*CA1* pyramidal neurons separately, as these subtrees have been reported to contribute to specific biophysical and computational properties in rodent *CA1* pyramidal neurons.^34,35,42-44^ We find that basal and oblique dendrites contribute equally to the total dendritic architecture and both compartments contribute significantly more compared to apical trunk + tuft (Figure 1I, inset, Kruskal-Wallis *p* < 0.001, Dunn’s post hoc test *p* < 0.01).

Oblique dendrites may be of particular importance to computational properties of individual neurons.^34,35,45^ We thus quantified the structural properties of basal vs. oblique dendrites in h*CA1* pyramidal neurons. We found that the number of primary trees is 3-fold larger for oblique dendrites compared to basal dendrites (*p* < 0.0001, Mann-Whitney test, Figure 1J). Oblique dendrites showed much less branching per tree compared to branching in basal dendrites (*p* < 0.0001, Mann-Whitney test, Figure 1K).

### I_h_ currents drive resonance at human theta oscillation frequencies

**Figure 2.**
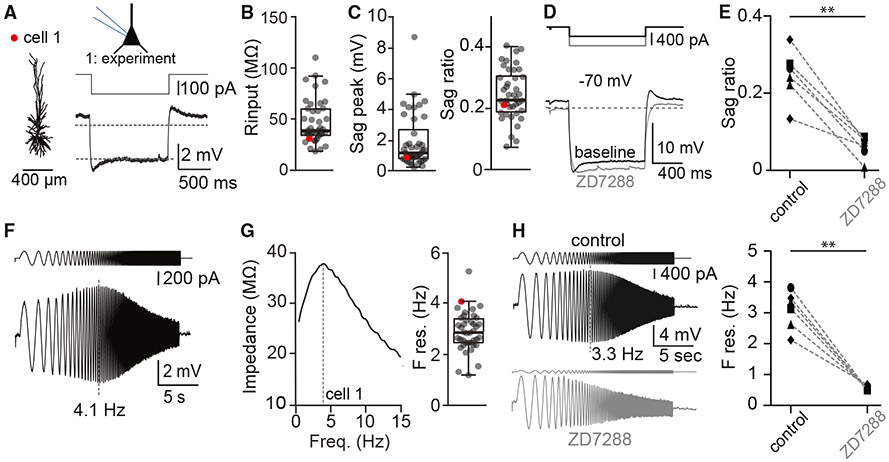
I_h_ currents drive resonance at human theta oscillation frequencies (A) Example h*CA1* pyramidal neuron with digital reconstruction of dendritic morphology and hyperpolarization current to uncover I_h_ (or sag current). Dashed lines correspond to −70 mV and steady-state hyperpolarization (close to −73 mV; see STAR Methods), respectively. (B) Population statistics for input resistance (38.8 MΩ, 33.7–58.9 [median, 1^st^–3^rd^ quartile range]). (C, left) Population statistics for sag peak (1.15 mV, 0.8–2.4 [median, 1^st^–3^rd^ quartile range]) and (C, right) sag ratio (0.23, 0.19–0.30 [median, 1^st^–3^rd^ quartile range]). (D) ZD7288 (HCN channel antagonist) blocks sag current during hyperpolarizing current injections (dark gray: control, light gray: ZD7288). (E) ZD7288 significantly reduces sag ratio across regions (*n* = 6, Wilcoxon signed-rank, *p* < 0.01). Symbols refer to entorhinal cortex (EC, ●), temporal cortex (T, ■), hippocampus (H, ♦), or frontal cortex (F, ▼). (F) Chirp protocol to extract resonance properties (see STAR Methods). (G, left) Impedance profiles of example cell #1 in response to the chirp input current. (G, right) Population statistics for preferred frequency (i.e., resonance frequency, F res., *n* = 40, 2.9 Hz, 2.5–3.4 [median, 1^st^–3^rd^ quartile range). (H, left) ZD7288 abolishes resonance properties in response to chirp protocol (black: control, gray: ZD7288). (H, right) ZD7288 significantly blocks resonance properties across regions (*n* = 6, Wilcoxon signed-rank, *p* < 0.01). Symbols are the same as in (E). Scale bar: 400 μm. See also Figure S2.

Given that no previous reports on whole-cell biophysical properties exist for non-pathological h*CA1* pyramidal neurons, we assessed basic neurophysiological properties of the neurons (Figure 2A). First, intrinsic resting membrane potentials were −63.4 mV (median; −66.2 to −60.1 mV, 1^st^–3^rd^ quartile range), and we observed spontaneous AP firing in only 3 out of 41 recordings (from *n* = 3 different slices and 2 surgical cases) and only immediately after establishing whole-cell configuration. No spontaneous APs were observed during subsequent protocols, confirming that the epileptic focus was not within our hippocampal slices. In addition, the input resistance was 37.8 MΩ (33.7–58.9 MΩ median, *n* = 41, 1^st^–3^rd^ quartile, Figure 2B), and the membrane time constant (tau) was 21.7 ms (median; 16.2–29.6 ms, 1^st^–3^rd^ quartile range, *n* = 41).

Rodent *CA1* pyramidal neurons show hyperpolarization-activated I_h_ currents, which endow them with resonance properties.^26,27,46^-^48^ This has never been tested in h*CA1* pyramidal neurons. We measured the voltage response to hyperpolarizing current injection to quantify the properties of I_h_ (Figure 2A), indicative of HCN channel activity. The amplitude of the hyperpolarizing current was scaled to the input resistance to consistently generate a hyperpolarization to approximately −73 mV. A sag response was found in all recordings (1.2 mV, median; 0.8–2.4 mV, 1^st^–3^rd^ quartile range, *n* = 41, Figure 2C, left). Normalization to the steady-state voltage response generates the dimensionless sag ratio, which was 0.23 (median; 0.19–0.30, 1^st^–3^rd^ quartile range, Figure 2C, right). We confirmed that this sag is due to HCN channel activation, as it was consistently blocked by the HCN channel antagonist ZD7288 (Figures 2D and 2E).

To test whether h*CA1* pyramidal neurons show resonance and, if so, at what frequency, we measured the voltage response to a chirp stimulus (Figure 2F; see STAR Methods; for mouse data, see Figure S2). When voltage response amplitudes showed a maximum at frequencies above 1 Hz, neurons were considered to be resonant (Figure 2G). Across recordings, we found that 40 out of 41 recorded neurons showed resonance frequencies above 1 Hz (98%). On the population level, the preferred frequency of h*CA1* pyramidal neurons was 2.9 Hz (median; 2.5–3.4 Hz, 1^st^–3^rd^ quartile range, *n* = 40, Figure 2G, right), with consistent values across surgical cases (F res. cases #1–#5, F res. 2.5 [*n* = 13], 2.8 [*n* = 3], 3.9 [*n* = 1], 3.2 [*n* = 15], and 2.8 Hz [*n* = 8]). Resonant properties were consistently abolished by the HCN channel antagonist ZD7288 across different regions (Figure 2H).

### Morpho-electric properties reveal cellular diversity along a multidimensional gradient

**Figure 3.**
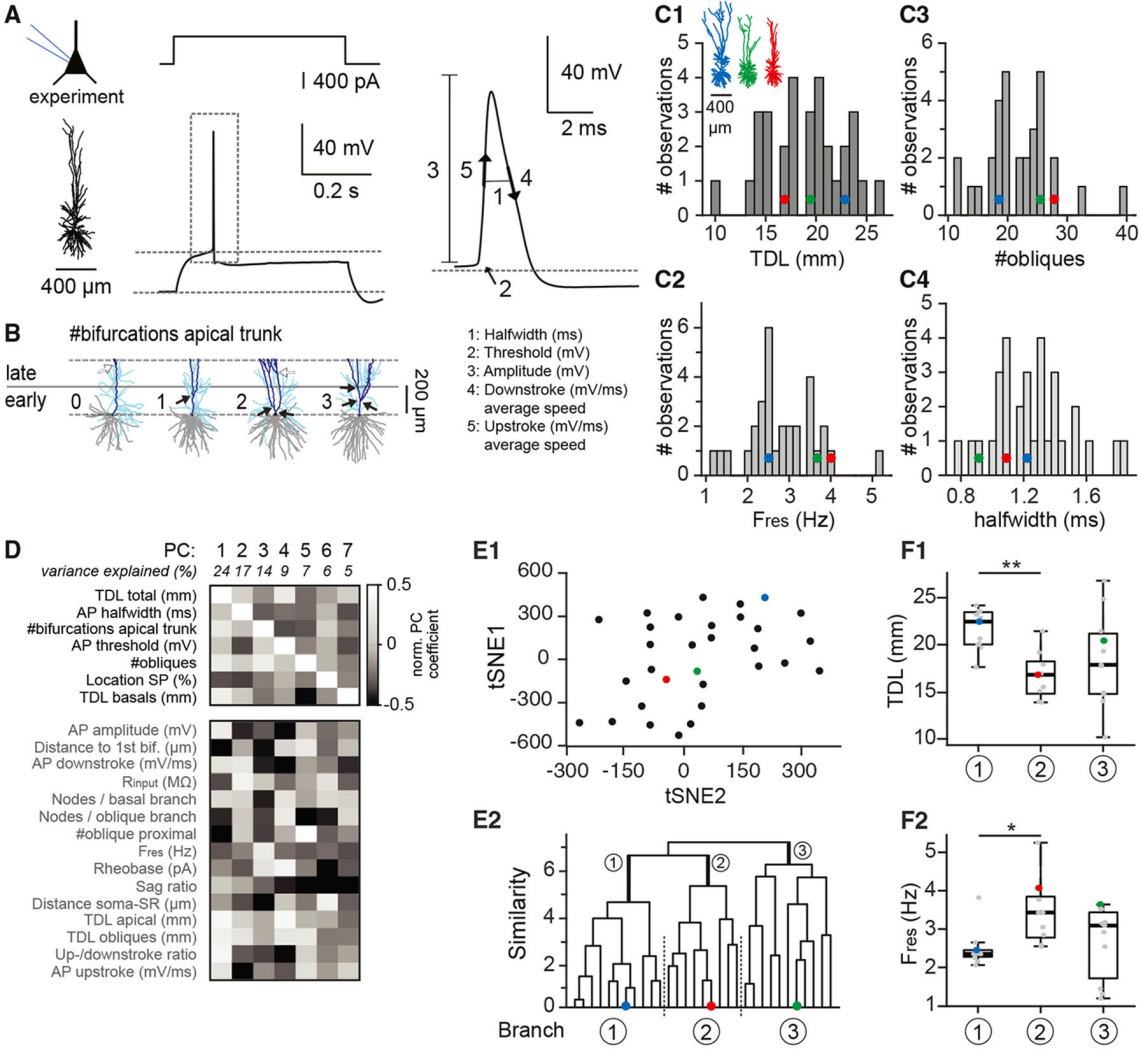
Morpho-electric properties reveal cellular diversity along a multidimensional gradient (A, left) Example morphology with matching positive current injection at rheobase. (A, right) Action potential (AP) features quantified. (B) Four example reconstructions showing “early” apical trunk branching within the first 200 μm of the main apical trunk (0, 1, 2, or 3 bifurcations, respectively [see also Benavides-Piccione et al.^38^). Note that neurons can also contain “late” bifurcations, located >200 μm from apical origin. (C1–C4) Distribution for a subset of features illustrating large single-parameter diversity within the population. Inset: three example morphologies. Bullets highlight their position in the histograms. Note that their position relative to each other is variable. (D) The morphological and electrophysiological features included for PCA. Gray values indicate the normalized PC coefficient for each individual feature vs. the PC (see STAR Methods). Features dominating the PCs are listed on top and ranked according to PC weight (white square). Additional features (listed below) are listed without weight. (E1) t-Distributed stochastic neighbor embedding (t-SNE) plot including the 22 morpho-electric features. Note the absence of clear, segregated clusters. (E2) Unsupervised hierarchical cluster analysis based on features in (D). Colored bullets represent example morphologies from (C1). (F1) TDL is significantly different for pyramidal neurons in branches 1 vs. 2 but highly diverse in branch 3. (F2) Resonance frequency is significantly different for pyramidal neurons in branches 1 vs. 2 but highly diverse in branch 3. Statistics: Kruskal-Wallis (unpaired, non-parametric ANOVA) with Dunn’s post hoc test, *p < 0.05 and **p < 0.01. All: *n* = 31. Boxplots illustrate median, 1^st^–3^rd^ quartile range. Scale bar: 400 μm. See also Figure S3.

To determine whether separate groups of h*CA1* pyramidal neurons with distinct morphological or biophysical properties could be identified, we analyzed a large morpho-electric parameter space of recorded pyramidal neurons. We used anatomical location of the soma along the dorsal-ventral axis of the *CA1 SP*, normalized as a fraction relative to the *SP-SLM* dimension to account for variability in *SP* thickness between surgical cases. Both passive properties (see Figure 2) as well as five active properties of APs were used, which were extracted from the first AP at rheobase (see STAR Methods; Figure 3A). Morphological measures included (among others) the TDL, number of obliques, number of nodes, and number of bifurcations in the first 200 μm of the apical tree (i.e., “early bifurcations,” Figure 3B).^38^

We quantified 12 morphological/anatomical and 10 electrophysiological parameters, which typically show a 2- to 6-fold range between minimum and maximal values (Figures 3C1 and 3C4). Principal-component analysis (PC) was performed on these 22 morpho-electric features (Figure 3D), and only PCs that explained more than 5% of the variance were included in the dataset. This generated 7 PCs, which together explain approximately 85% of the variance. We subsequently determined which parameters contributed most to the 7 PCs, reflected as the PC coefficient normalized to the feature with maximal contribution (Figure 3D). Among the 7 morpho-electric features that contribute most to the PCs, we did not find pairwise correlations (Spearman correlations, not significant after correction for multiple testing).

The t-distributed stochastic neighbor embedding plot based on all 22 morpho-electric features does not identify segregated clusters (Figure 3E1) but rather a gradient along a morpho-electric multiparameter space. Unsupervised hierarchical cluster analysis on all features in combination with gap statistic^49^ to estimate the number of clusters suggest a single cluster with three main branches of h*CA1* pyramidal neurons (Figures 3E2 and S3; see STAR Methods). Five out of seven PCs do not show significant differences between branches (Kruskal-Wallis, *p* > 0.05). We find that TDL (primarily contributing to PC1; see also Figure 1) is significantly different between branches 1 and 2 but highly variable for branch 3. TDL of only the basal dendrites (PC7) shows significantly lower values for branch 2 compared to branches 1 and 3 (Figure S3). Finally, resonance frequency does not strongly contribute to any of the 7 PCs but is significantly different between branches 1 and 2 and highly variable for branch 3. To conclude, we find a single cluster with cellular diversity along a multidimensional gradient rather than multiple cell types with clearly distinguishable morpho-electric properties.

**Figure 4.**
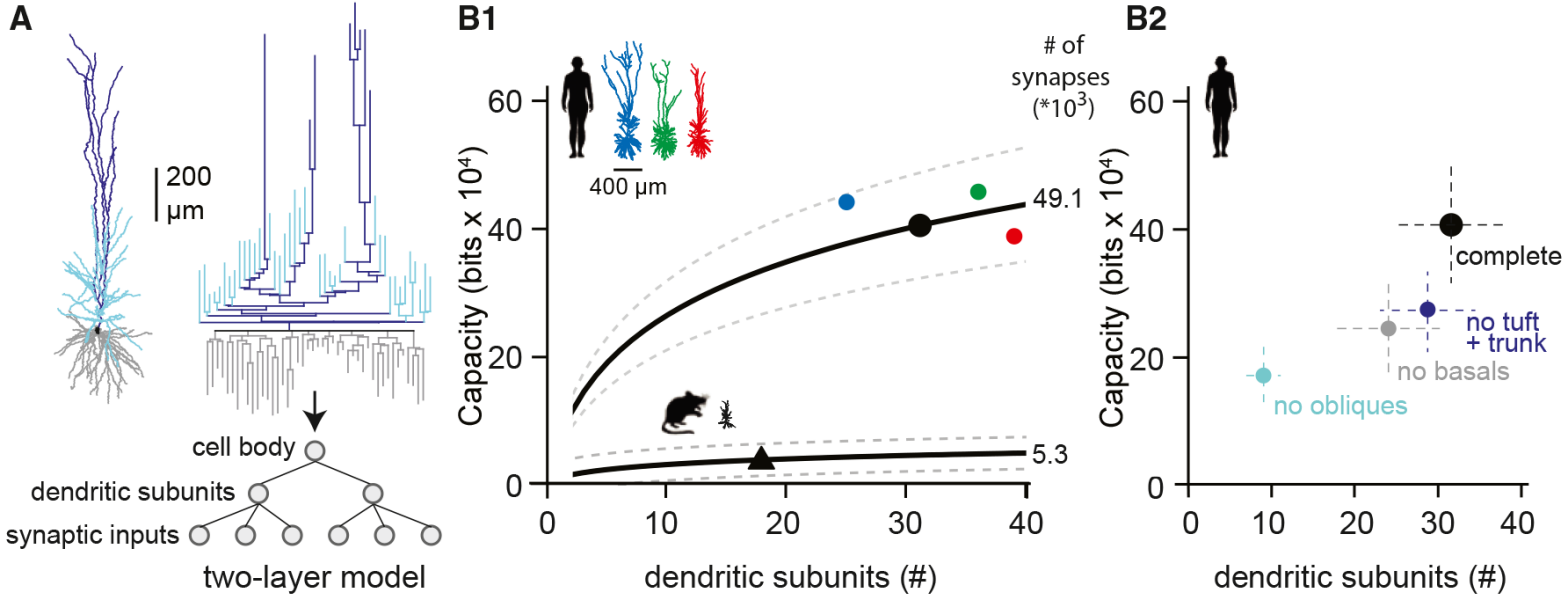
Structural complexity supports memory capacity (A) Example morphology (same as Figures 2 and 3) and associated structural dendrogram. (B1) Average memory capacity of h*CA1* and mouse *CA1* pyramidal neurons when considered as a two-layer model. Bracket lines represent standard deviation, and colored bullets represent the three example morphologies. Note: memory capacity of h*CA1* pyramidal neurons is 11-fold higher compared to mouse due to branching characteristics and increased numbers of dendritic subunits and spine count (synapses). (B2) Impact of oblique and basal dendritic trees on memory capacity of h*CA1* pyramidal neurons. Note that the contribution of oblique dendrites to number of subunits and memory capacity exceeds that of basal dendrites (bullets illustrate mean, bracket lines standard deviation). Human: *n* = 31, mouse: *n* = 5. See also Figure S4.

We showed that h*CA1* pyramidal neurons are characterized by large morphologies and many oblique dendrites (Figure 1). To translate this structural geometry into memory capacity, we used the two-layered model put forward by Poirazi and Mel.^50^ We use the number of primary trees (basal trees originating from the soma and number of oblique dendrites originating from the apical trunk) as a measure for the number of independent units plus number of apical trees after early bifurcations. We thus assume that primary dendritic trees (not branches) operate as independent units (but see Losonczy and Magee^34^), and therefore our definition of the neuron’s complexity may provide a lower bound for the memory capacity (Figure 4A). Our analysis also assumes non-linear electrical properties of dendrites, which is based on experimental evidence from both rodent hippocampus literature^34,43,51-53^ and dendritic recordings from human (cortical) pyramidal neurons.^54-58^

We find that the population of human pyramidal neurons has on average 31 ± 6 (average ± SD) subunits and memory capacity of h*CA1* pyramidal neurons exceeds that of mouse *CA1* pyramidal neurons by an order of magnitude (11.3-fold difference, Figure 4B1; see also Figure S4). Removing the large number of primary oblique dendrites and associated synaptic inputs causes the total memory capacity to decrease to 42%, which is a larger step compared to removing basal dendrites and its synapses (60% remaining) or removing the apical tree and its synapses (68% remaining). Thus, even though the TDLs for basal and oblique dendrites are comparable (Figure 1), the specific structural characteristics (Figures 1J and 1K) translate to compartment-specific contribution to memory capacity (Figure 4B2). These calculations are based on conservative settings for number of subunits (m: dendritic trees) and number of independent presynaptic partners (d = s/5). It is unknown how these assumptions align to the biological reality, but less conservative settings for number of independent subunits (m: terminal branches) and number of independent presynaptic partners (d = s/1.2) translate to augmented memory capacity of h*CA1* and mouse *CA1* pyramidal neurons (Figure S4).

To conclude, we quantified the morpho-electric properties and memory capacity of human hippocampal *CA1* pyramidal neurons. We show large cellular diversity, consistent resonant properties at low theta frequencies, and extended morphologies, which translate to powerful memory capacity.

## DISCUSSION

The hippocampal formation is responsible for evolutionary conserved behaviors such as spatial navigation, learning, and memory encoding/consolidation. These cognitive functions rely on passive and active biophysical properties of pyramidal neurons that were never characterized in human hippocampus. Here, we used acute resection samples of non-pathological human hippocampus and uncovered structural and biophysical characteristics of these cells. We show that (1) h*CA1* pyramidal neurons have extended dendritic architecture and many oblique dendrites (Figure 1), (2) neurons consistently show resonant properties, and the preferred frequency (Figure 2) corresponds to the lower theta frequencies recorded in human subjects, (3) single morpho-electric parameters cover a broad range with no interdependence, resulting in a gradient of cellular diversity (Figure 3), and (4) memory capacity is larger in human relative to mouse (Figure 4). Thus, h*CA1* pyramidal neurons differ from mouse *CA1* pyramidal neurons in all properties studied, including increased structural complexity and enriched memory capacity.

The resonance frequency of h*CA1* pyramidal neurons (2.9 Hz) we uncovered in single-neuron recordings closely matches the *in vivo* theta rhythm of human hippocampus (i.e., 1–4 Hz).^17,19,59^ The temporal domain at which the human hippocampus operates is thus slower compared to rodents, as theta rhythms in mice and rats occur at higher frequencies (i.e., 4–10 Hz).^2,20,60^ We found that the preferred frequencies of h*CA1* (2.9 Hz) and mouse *CA1* (2.9 Hz) pyramidal neurons are highly comparable (Figures 2 and S2). This indicates that theta rhythm in mice (4–10 Hz) and preferred frequency of mouse *CA1* pyramidal neurons (2.9 Hz) do not match. In contrast, theta in human (1–4 Hz) and preferred frequency in h*CA1* pyramidal neurons (2.9 Hz) overlap. Furthermore, while nearly all neurons had resonant properties in humans, a large fraction of neurons were non-resonant in mice.

In rodent *CA1* pyramidal neurons, it was shown that synaptic activity in individual dendritic branches triggers local NMDA spikes.^42,43^ This could support branch-selective integration of synaptic inputs^34,35^ or branch-constrained synaptic plasticity.^61^ When multiple dendritic branches are activated simultaneously, the more global depolarization can be sufficient to activate dendritic voltage-dependent calcium channels (i.e., trigger a Ca^2+^ spike), resulting in high-frequency bursts at the soma.^43,53,62,63^ The dendritic architecture of h*CA1* pyramidal neurons could facilitate such compartmentalization and non-linearities, but this remains completely unknown at present. Without dendritic recordings, it also remains unknown how extended h*CA1* morphologies relative to mouse scale in the context of dendritic function.^58^ This includes occurrence of local NMDA spikes, dendritic Na^+^ spikes, Ca^2+^ transients, and dendritic compartmentalization.^42,43,53,57,62^

We found large diversity in morpho-electric properties (Figure 3). This is important in view of cellular diversity in rodent *CA1*, which has several axes, including transcriptional profile,^64^ preferred theta phase,^65^ spiking properties,^52,66^ strength of AP backpropagation,^67^ or neuronal properties associated with dorsal/ventral soma location.^28^ Based on genetic profiling in the human hippocampus, two major classes of pyramidal cell types were put forward in the hippocampus,^68,69^ so an obvious way forward would be to determine the overlap between morphoelectric diversity and transcriptional cell types.

Across species, *CA1* pyramidal neurons consistently show a large number of oblique dendrites.^31,32,46^ These oblique dendrites show highly specialized properties including integrative properties,^33-35^ excitability,^42^ and signal propagation.^33,70^ Since individual oblique dendrites may represent an additional site for AP or NMDA spike generation,^71^ the large number of oblique dendrites in h*CA1* pyramidal neurons may boost the memory (and computational) capacity of these neurons.^34,50,72^ Such computational capabilities are expected to be dramatically lower for rodent *CA1* pyramidal neurons, since the architecture of the apical dendrite does not show similar topology.^38^

### Limitations of the study

Average resonance frequency was remarkably consistent across subjects. This may be surprising considering divergent demographics for surgical cases such as age, genetic background, or disease history but could also imply this feature is a hard-wired, fundamental intrinsic property of h*CA1* pyramidal neurons. We only included hippocampal specimen without evident structural alterations but cannot ignore that the surgeries are performed to treat brain pathology and associated epileptic seizures (Table S1). It would require much larger datasets to determine true biological variability or variability that emerged due to disease history or anti-epileptic medication, but these type of surgical resection samples (and associated datasets) are extremely sparse. It is promising that larger datasets on human resection tissue failed to reveal a link between disease history and morpho-electric properties.^73-75^

Furthermore, we explored cellular diversity for single parameters, pairwise correlations, and full morpho-electric parameter space. We excluded digital reconstructions with major truncation artifacts, but it is impossible to capture the full dendritic architecture of these extended morphologies in 350 μm thin brain slices.^76^ The truncation varies from slice to slice, especially for distal dendrites in the *SR* and tuft dendrites in the *SLM*. We also only captured electrical properties using somatic patch-clamp recordings and do not know how truncation artifacts may have had an impact on our somatic recordings. In general, the current analysis on cellular diversity may benefit from multimodal datasets driven by transcriptomics to map the cellular constituents and associated morpho-electric cellular diversity of the h*CA1* microcircuit.^74,77,78^ These larger datasets should ideally include dendritic recordings to validate our assumptions on independent dendritic subunits and non-linear properties (Figure 4).

To conclude, we show that h*CA1* pyramidal neurons have elaborate dendritic trees and morpho-electric properties, which translate to a boost in memory capacity. h*CA1* pyramidal neurons show a clear frequency preference, which is consistent across neurons and subjects. The ability of h*CA1* pyramidal neurons to respond to a preferred frequency causally depends on HCN channel function,^79^ and the preferred frequency accurately matches the hippocampal theta rhythm observed during complex human behaviors. The combination of deep brain recording techniques^80,81^ and single-cell recordings in non-sclerotic human resection tissue for transcriptomic classification of cell types^74^ paves an exciting way to uncover additional unknown aspects of human hippocampus function including genes, cells, circuits, and ultimately cognitive behavior.

## STAR★METHODS

### RESOURCE AVAILABILITY

#### Lead contact

Further information and requests for resources and reagents should be directed to and will be fulfilled by the lead contact, Christiaan de Kock (ckock@falw.vu.nl).

#### Materials availability

This study did not generate new unique reagents.

#### Data and code availability

##### Data:

Morphological reconstructions and raw electrophysiology reported in this paper will be shared by the lead contact upon request. Data of figures have been deposited at DataverseNL and are publicly available as of the date of publication. Accession numbers are listed in the key resources table.

##### Code:

Analysis code has been deposited at Github and is publicly available as of the date of publication, as seen in the key resources table.

##### Additional information:

Any additional information required to reanalyze the data reported in this paper is available from the lead contract upon request.

### EXPERIMENTAL MODEL AND STUDY PARTICIPANT DETAILS

#### Human surgical specimens

All procedures were performed with the approval of the Medical Ethical Committee of the VU medical center (VUmc), and in accordance with Dutch license procedures and the Declaration of Helsinki. All patients provided written informed consent.

Data included in this study were exclusively obtained from (non-pathological) neurosurgical tissue resections for the treatment of temporal lobe epilepsy (n = 4) or epilepsy with tumor (n = 1) or unknown treatment (n=1) in 4 male and 2 female patients (Table S1). We did not detect any influence of gender on the morphological/physiological properties.

#### Mouse specimens

All procedures involving mice were approved by the animal ethical care committee of the VU university. Mixed strains of male (n=2) and female (n=2) mice from 63-70 days old were used for experiments. Mice were maintained on a 12 h light/dark cycle in a temperature and humidity-controlled room. Mice were housed 3-6 per cage with free access to food and water. We did not detect any influence of gender on the morphological/physiological properties.

### METHOD DETAILS

#### Human acute brain slice preparation

During surgical treatment of underlying brain pathology, the hippocampus was taken out “en block”, in addition to (partial) resection of the temporal lobe. Structural integrity of resected hippocampus was assessed with 1) presurgical MRI, 2) assessment of the cytoarchitectural integrity by an expert pathologist, 3) differential interference contrast images during electrophysiology, and finally 4) post-hoc histology of tissue used for electrophysiology (NeuN and biocytin-DAB). We obtained hippocampal tissue from a total of 6 patients: 5 patients from the VU Medical Center (VUmc) and 1 patient from Harborview Medical Center (Table S1). In these hippocampal specimens, presurgical MRI and posthoc histology did not reveal structural abnormalities (Table S1).

Immediately upon resection, the tissue block containing hippocampus proper was placed into a sealed container filled with ice-cold, artificial cerebral spinal fluid (aCSF). For one sample, initial aCSF consisted of (in mM): 110 choline chloride, 26 NaHCO_3_, 10 D-glucose, 11.6 sodium ascorbate, 7 MgCl_2_, 3.1 sodium pyruvate, 2.5 KCl, 1.25 NaH_2_PO_4_ and 0.5 CaCl_2_. For five additional samples, aCSF consisted of (in mM): 92 *N*-methyl-d-glucamine chloride (NMDG-Cl), 2.5 KCl, 1.2 NaH_2_PO_4_, 30 NaHCO_3_, 20 4-(2-hydroxyethyl)-1-piperazineethanesulfonic acid (HEPES), 25 d-glucose, 2 thio-urea, 5 sodium-l-ascorbate, 3 sodium pyruvate, 0.5 CaCl_2_.4H_2_O and 10 MgSO_4_.7H_2_O. Before use, the aCSF solution was carbogenated with 95% O_2_, 5% CO_2_ and the pH was adjusted to 7.3 by addition of 5M HCl solution, after which it was put on ice until use.

The transition time between resection in the operation room and arriving at the neurophysiology lab was < 20 minutes. Immediately upon arrival at the lab, slice preparation commenced. First, residual blood was rinsed from the tissue block, while remaining submerged in ice-cold NMDG solution. Next, orientation of the tissue for slicing was determined based on gross macroscopic anatomical hallmarks of the hippocampus proper and adjacent structures.

Next, the tissue block was glued onto the slicing platform such that the slice angle ensured optimally intact (apical) dendrites, and 350-μm-thick coronal slices of the hippocampal body were prepared using a vibratome (Leica V1200S), in ice-cold aCSF solution. Each slice was then transferred to a warmed holding chamber (34°C), containing NMDG-based aCSF, for 12 minutes under constant carbogenation. Next, slices were transferred to a holding chamber with aCSF containing (in mM): 92 NaCl, 2.5 KCl, 1.2 NaH_2_PO4, 30 NaHCO_3_, 20 HEPES, 25 d-glucose, 2 thiourea, 5 sodium-L-ascorbate, 3 sodium pyruvate, 2 CaCl_2_.4H_2_O and 2 MgSO_4_.7H_2_O (pH 7.3) and stored at room temperature for at least an hour until used. The time between tissue resection and electrophysiological recordings could thus vary between 2 - 15 hours. Osmolarity of various aCSF solutions were set to 310 mOsm, using the Vapro (5600 Vapor pressure, Elitech) osmometer.

#### Mouse acute brain slice preparation

All procedures related to mice were approved by the animal ethical care committee of the VU university. Wild-type C57BL/6J mice (n = 4, age 63-70 days, 2 males, 2 females) were anaesthetized with euthasol (i.p., 120 mg/kg in 0.9% NaCl), and transcardially perfused with 10 mL ice-cold carbogen-saturated NMDG solution. Upon removal of the brain, 350 μm thick coronal slices were obtained as described above.

#### Electrophysiology

Recordings were obtained at 34°C, in aCSF containing (in mM): 125 NaCl, 3 KCl, 1.2 NaH_2_PO_4_, 1 MgSO_4_, 2 CaCl_2_, 26 NaHCO_3_, and 10 d-glucose (310 mOsm), continuously bubbled with 95% O_2_, 5% CO_2_. In a subset of experiments, recordings were made in aCSF containing (in mM): 126 NaCl, 2.5 KCl, 1.25 NaH2PO4, 26 NaHCO3, 12.5 glucose, 2 CaCl2·4H20,1 MgSO4·7H2O,1 Kynurenic acid and 0.1 picrotoxin (pH 7.3). Borosilicate glass patch pipettes (3-6 MΩ resistance) (Harvard apparatus/Science products GmbH) were filled with intracellular solution, containing (in mM): 115 K-gluconate, 10 HEPES, 4 KCl, 4 Mg-ATP, 10 K-Phosphocreatine, 0.3 GTP, 0.2 EGTA, and biocytin 5 mg/ml, pH adjusted to 7.3 with KOH, osmolarity 295 mOsm/kg). For patch-seq experiments, internal solution contained (in mM): 110.0 K-gluconate, 10.0 HEPES, 0.2 EGTA, 4 KCl, 0.3 Na2-GTP, 10 phosphocreatine disodium salt hydrate, 1 Mg-ATP, 20 μg/ml glycogen, 0.5U/μL RNAse inhibitor (Takara, 2313A), 0.5% biocytin and 0.02 Alexa 488. The pH was adjusted to 7.3 with KOH, osmolarity to 295 mOsm/kg.

Whole-cell patch-clamp recordings were made with a Multi-Clamp 700B (Molecular Devices), digitized at a rate of 20 kHz (ITC-18 computer interface instrutech corporation / National Instruments USB-6343) and obtained with either PClamp 10 (Molecular Devices) or MIES (Allen institute) in IgorPro 8 (WaveMetrics). Files were stored in Axon Binary File (abf) and Neurodata Without Borders (nwb) format, respectively. Prior to recording the pipette offset was compensated for, and in current clamp the bridge was balanced. Pipette access resistance was monitored throughout the recording, and was between 5-18 MΩ. Recordings with a pipette access resistance >20 MΩ were excluded from the dataset. HCN channel antagonist ZD7288 (10 μM; Hello bio) or voltage-gated calcium channel blocker cadmium (CdCl_2_) (10 μM; J.T. Baker) were prepared in recording aCSF, and washed in upon completion of all protocols, after which the protocols were repeated.

#### Immunohistochemistry

After recordings, slices were fixed in 4% paraformaldehyde (in phosphate buffer) for a minimum of two days. Subsequently, biocytin-filled neurons were recovered using the chromogen 3,3-diaminobenzidine (DAB, Vectastain) tetrahydrochloride avidin–biotin–peroxidase method. Both at the department of (Neuro)Pathology at the UMC and our lab, the neurotypical status of the tissue was evaluated using a staining for Neuronal Nuclei (NeuN) using rabbit-anti-NeuN primary antibody (1:1000, ThermoFisher, catalog number PA578499) and Biotinylated Affinity Purified Goat Anti-Rabbit IgG secondary antibody (1:225, ThermoFisher, 32054) for a subset of tissue samples. Slices were mounted on slides and embedded in mowiol (Clariant) or Aqua-Poly/Mount (Polysciences).

### QUANTIFICATION AND STATISTICAL ANALYSIS

#### Neurophysiology

We applied multiple stimulation protocols to obtain passive and active (sub- and suprathreshold) properties of the human *CA1* pyramidal neurons. All protocols started at an imposed membrane potential of −70 mV ± 1 mV.

##### Passive properties

To assess passive subthreshold properties, hyperpolarizing square 1s current injections were applied, starting from −150 pA, with increments of +25 pA. A −25 pA square current injection was used to calculate the input resistance and membrane time constant (tau). HCN channel activity was quantified on sweeps with current injections leading to 3 mV hyperpolarization (to compensate for the difference in response between recordings due to input resistance variability). The difference between sag peak and steady-state hyperpolarizing responses was defined as delta sag peak (in mV). To generate the sag ratio, the delta sag peak was divided by the steady state hyperpolarization response of the same sweep.

Next, resonance frequency (Figure 2) was determined from the voltage response (± 5-10 mV) to a sinusoidal current injection with a frequency range of 0.5 to 30 or 40 Hz (in 10 or 20 s, respectively). Next, the impedance amplitude profile (ZAP) was derived via the ratio of the fast Fourier transform of the voltage response to the fast Fourier transform of the sinusoidal current injection. The resonance frequency (F_res_) reflects the frequency at which maximum impedance was observed.

##### Active properties

To obtain active, suprathreshold membrane properties, a 1s depolarizing square current injection was applied, ranging from +25 pA to 1000 pA, with increments of 25 pA. The smallest current injection that resulted in an action potential (AP), was set as the rheobase value, and this first sweep exceeding AP threshold was used for all analyses of active suprathreshold membrane properties (AP threshold, AP amplitude, AP upstroke, AP downstroke and AP halfwidth). AP threshold was defined as the point where the slope exceeded 23 mV/ms. AP amplitude was specified as the voltage change between the AP threshold and the AP peak. AP halfwidth was defined as the width of the AP (in ms) at half maximal amplitude. Upstroke and downstroke speed (mV/ms) are computed as the mean rising and fall speed between 30 and 70% of the AP, respectively.

##### Control for burst spiking

Burst spiking was not part of the features as we typically encountered a single spike (26 out of 41 recordings) upon rheobase stimulation or multiple spikes at low frequencies (3.2 Hz, 2.1 - 6.3 Hz, median, 1st - 3rd Quartile range, n = 15). We found only n = 1 pyramidal neuron with instantaneous frequency of 73 Hz at rheobase current injection, but this is still considered regular spiking.^52^ At maximal current injection, only n = 2 neurons exhibit an instantaneous frequency exceeding 100 Hz (interspike interval (ISI) cell 1 = 9.4 ms, ISI cell 2 = 9.5 ms) and could be considered bursting neurons, but only upon maximal current injection. Upon short, 3 ms current injections, human *CA1* pyramidal neurons are capable of spiking at high frequencies (> 100 Hz, see below) and we thus conclude that prolonged somatic current injection does not favor burst spiking in our human resection samples.

#### Morphological reconstruction

All recovered neurons underwent critical quality assessment of staining quality and occurrence of slicing artifacts. Only neurons that passed quality control were reconstructed with Neurolucida software (Microbrightfield, Williston, VT, USA), using an 100x oil objective. Using DAB and NeuN histology, *Stratum Pyramidale (SP)* could be unambiguously identified, as well as the border between *SP* and *Stratum Radiale* (*SR*). The border between *SR* and *Stratum Lacunosum Moleculare (SLM)* was estimated based on differences in contrast for *SR* and *SLM* layers using light microscopy. Finally, reconstructed *CA1* neurons were annotated relative to layer borders.

#### Statistical analysis

Data were analyzed using analysis scripts written in Matlab 2021A (MathWorks) and statistical analyses were performed using Prism 7.2 (GraphPad Software). All electrophysiological analyses were performed using customized Matlab scripts (Source code available at https://github.com/ElineJasmijn/Morphys, taken and adjusted from https://github.com/INF-Rene/Morphys), see key resources table. Memory capacity analyses were performed with a customized python script, see key resources table. Non-parametric data are visualized in boxplots (generated in Matlab 2021a) with the central mark as the median, the edges of the box the 25th and 75th percentiles, the whiskers extending to the most extreme data points, excluding the outliers. In all boxplots, each dot represents a single morphology or recording. Parametric data are visualized in histograms illustrating average and standard deviation. All variables were tested for normality. The ANOVA test was used for normally distributed data. The Mann-Whitney test was applied when comparing two independent, non-normally distributed data samples. For three independent samples the Kruskal-Wallis test was used in combination with the Dunn’s post-hoc test. The Wilcoxon test was applied for non-parametric paired samples. All corresponding p values and numbers of samples (i.e. individual recordings) are present in relevant figure legends. Univariate statistical tests were performed on Figures 1, 2, 3, S1, and S2. In Figure 1, we compared morphological features; In Figure 2, we compared resonance frequency with and without a HCN channel antagonist; in Figure 3, we compared morpho-electric properties related to dendrogram structure; in Figure S1, we compared total dendritic length across species and used the mean to compare the effect size and standard deviation as a measure of the spread (shown in error bars); in Figure S2, we compared electrophysiological features between human and mouse and the fraction of resonant pyramidal cells for human versus mouse (Fisher exact test); in Figure S3, we compared additional morpho-electric properties related to dendrogram structure. For dimensionality reduction, we used the tsne and dendrogram packages in Matlab 2023b.

#### Cluster analysis

We used 22 structural and biophysical features from n = 31 digital reconstructions and electrophysiological recordings and this subset with bimodal data originated from 3 patients. To reduce the dimensionality of the dataset and identify the most informative features for clustering, we performed a principal component analysis (PCA).^82^ Only principal components that explained more than 5% of the variance were included in the dataset, resulting in a total of 7 principal components, which together explained approximately 83.6% of the variance. We next used unsupervised hierarchical clustering and clustering results were visualized using a 2-dimensional dendrogram and t-SNE plot (Figure 3).

Pairwise comparisons were performed for the 7 morpho-electric features dominating the principal components. We excluded the correlation between total dendritic length (TDL) and TDL basal dendrites, as TDL basal dendrites is embedded in TDL. Corrected *p*-value for multiple testing was set to: 0.05/20 correlations = 0.0025.

To estimate the number of clusters, we computed the gap statistic criterion for hierarchical clustering using 2000 reference null distributions with the MATLAB function evalclusters.^49^ The maximum value of the gap statistic indicates the estimated number of clusters in the data (here: “1”).

#### Calculation of memory capacity

Memory capacity of hCA1 neurons was computed using the formula: ^50^

(Equation 1) $$B_{n}(m,k,d)=2\cdot \log\left(\begin{array}{c} \left(\begin{array}{c} k+d-1\\ k \end{array}\right)+m-1\\ m \end{array}\right)$$

where m is the number of dendritic subunits, s is the total number of synapses, d the number of distinct afferent input lines (d = s/5) and k is s/m, the number of synapses in each subunit. The number of distinct afferent input lines is constrained by the number of synapses between pairs of pyramidal neurons. These data are not available for human pairs of *CA1* pyramidal neurons. For rodent *CA1*, the experimentally estimated number of synapses per connection is 1.2 ± 0.4.^36,83^ This is lower compared to the first-approximate estimation based on axonal-dendritic overlap of morphologies and connectivity statistics (i.e., 5 contacts^84^). For pairs of human cortical L2/L3 pyramidal neurons, the upper limit of putative synapses is 5.^85^ In line with conservative constraints for independent subunits (trees, not branches), we used the upper limit of putative synapses (n=5 per connection) to generate a conservative number of independent presynaptic partners.

Memory capacity was determined for both *hCA1* and *mCA1* pyramidal neurons. Total number of synapses was the product of total dendritic length (Figure 3) and branch-specific spine density^38^ (Table S2). The number of dendritic subunits was estimated (a lower bound) as the sum of the number of primary basal dendrites emerging from the soma, the number of oblique dendrites emerging from the apical trunk and the number of main apical trees (as a derivative of # of early apical tree bifurcations). We assumed that these subunits represent independent compartments,^34,43,51-53^ and capable of nonlinear input-output transformations.^54-58^ For mouse we determined total dendritic length of n = 5 representative morphologies from^38^ and multiplied these values with branch-specific spine density to compute total number of synapses (s = 5312,^86^). In addition, m = 17 is based on the same n = 5 representative morphologies. Finally, d = s/5.

For Figure S4, the number of terminal branches represent the number of independent subunits (“m”) and the number of independent presynaptic partners was calculated as d = s/1.2.

## Supplementary Material

1

2

## Figures and Tables

**Table:** KEY RESOURCES TABLE

REAGENT or RESOURCE	SOURCE	IDENTIFIER
Antibodies		
NeuN Polyclonal Antibody	ThermoFisher	PA-578499. RRID:AB_2736206
Chemicals, peptides, and recombinant proteins		
ZD7288	Hello bio	HB1152
Cadmium	J.T.Baker	N/A
Biocytin	Molekula	36219518
Mowiol	Clariant GmbH	N/A
Aqua-Poly/Mount	Polysciences	N/A
Alexa 488	ThermoFisher	A10436
RNAse inhibitor	Takara	2313A
NMDG-Cl	Sigma-Aldrich	24896593
Critical commercial assays		
VECTASTAIN^®^ Elite^®^ ABC-HRP Kit	Vectorlabs	PK-6100. RRID:AB_2336819
ABC peroxidase Rabbit IgG staining kit	ThermoFisher	32054
Deposited data		
Data repositories for each figure	This study.	https://doi.org/10.34894/VJDRZC
Experimental models: Organisms/strains		
Human neurological samples	Table S1	N/A
Mouse	Charles River	C57BL/6J
Software and algorithms		
Igor Pro	Wavemetrics	RRID: SCR_000325: https://www.wavemetrics.com/products/igorpro
MIES	Allen Institute	RRID:SCR_016443: https://github.com/alleninstitute/mies
PClamp 10	Molecular devices	RRID:SCR_011323: http://www.moleculardevices.com/products/software/pclamp.html
Neurolucida	MBF bioscience	RRID:SCR_001775: https://www.mbfbioscience.com/products/neurolucida
Neurolucida Explorer	MBF bioscience	RRID:SCR_017348: https://www.mbfbioscience.com/neurolucida-explorer
Matlab	Mathworks	RRID:SCR_001622: https://mathworks.com
Matlab analysis code	This study	https://doi.org/10.5281/zenodo.10813442
Python	Python.org	RRID: SCR_008394
Python analysis code	This study	https://doi.org/10.5281/zenodo.10813554
Prism 7.2	Graphpad Software	RRID:SCR_002798: https://www.graphpad.com/
Illustrator	Adobe	RRID:SCR_010279: http://www.adobe.com/products/illustrator.html
Other		
vibratome	Leica V1200S	N/A
Multi-Clamp 700B	Molecular Devices	N/A

## Highlights

- Human *CA1* pyramidal neurons show extended morphologies compared to mouse *CA1* neurons
- HCN channel activity supports cellular resonance at low theta frequencies
- Morpho-electric analysis indicates cellular diversity along a multidimensional gradient
- Memory capacity of human *CA1* pyramidal neurons is much larger compared to mouse *CA1* neurons
