# Supplementary material for: Morpho-electric diversity of human hippocampal CA1 pyramidal neurons
Source: Cell Rep. Author manuscript; Available in PMC 2024 May 21. (PMC11106460; doi:10.1016/j.celrep.2024.114100)
Supplement: 1 [file NIHMS1988720-supplement-1.pdf]

Cell Reports, Volume *43*

Supplemental information

Morpho-electric diversity of human hippocampal *CA1* pyramidal neurons

Eline J. Mertens, Yoni Leibner, Jean Pie, Anna A. Galakhova, Femke Waleboer, Julia Meijer, Tim S. Heistek, René Wilbers, Djai Heyer, Natalia A. Goriounova, Sander Idema, Matthijs B. Verhoog, Brian E. Kalmbach, Brian R. Lee, Ryder P. Gwinn, Ed S. Lein, Eleonora Aronica, Jonathan Ting, Huibert D. Mansvelder, Idan Segev, and Christiaan P.J. de Kock

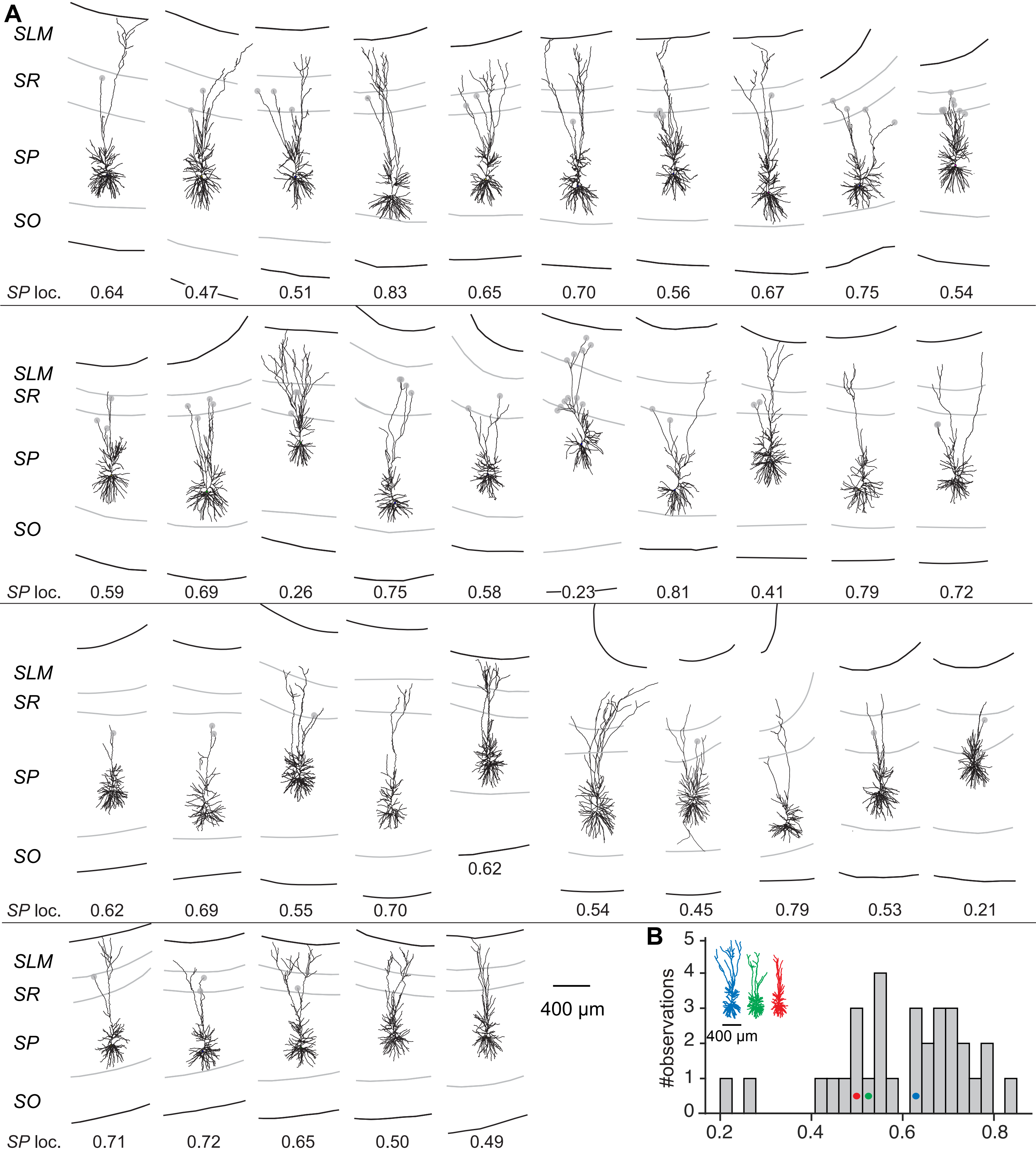

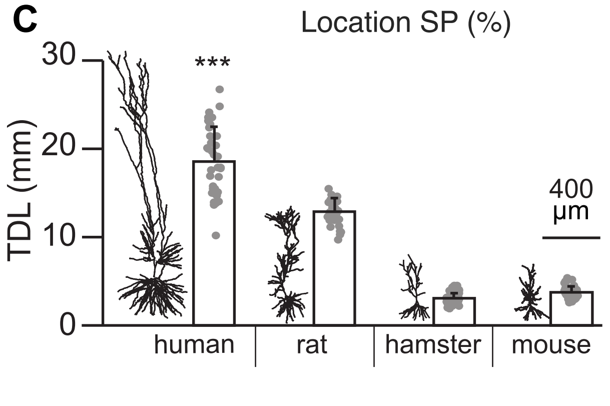

Fig.S1: Gallery of n = 35 human CA1 pyramidal neurons used for morphological analysis (Fig.1) and cellular diversity analysis (subset of n = 31 with morphology and electrophysiology), annotated according to layer position. A) For the n = 31, each of the electrophysiological features used for cluster analysis was mapped. SP: Stratum Pyramidale, SO: Stratum Oriens, SR: Stratum Radiatum, SLM: Stratum Lacunosum Moleculare. The value "fraction in SP" refers to the position of the cell body within the SP according to the measure "(distance Soma - SLM)/(distance SLM - SO)". Cluster identity (in color code matching Fig.3) is provided below each reconstruction. NA: not applicable (morphologies not included in cluster analysis). Slicing artefacts of the apical dendrite are indicated by grey bullets, superimposed on the truncated dendritic branch. B) Distribution of relative soma location in SP. C) Total dendritic length (TDL) of human CA1 pyramidal neurons significantly exceeds TDL in rat, hamster, and mouse (ANOVA, $p < 0.001$). For rodent data, see Neuromorpho.org and (1-4). Related to Figures 1 and 3.

Mertens et al, Fig.S1

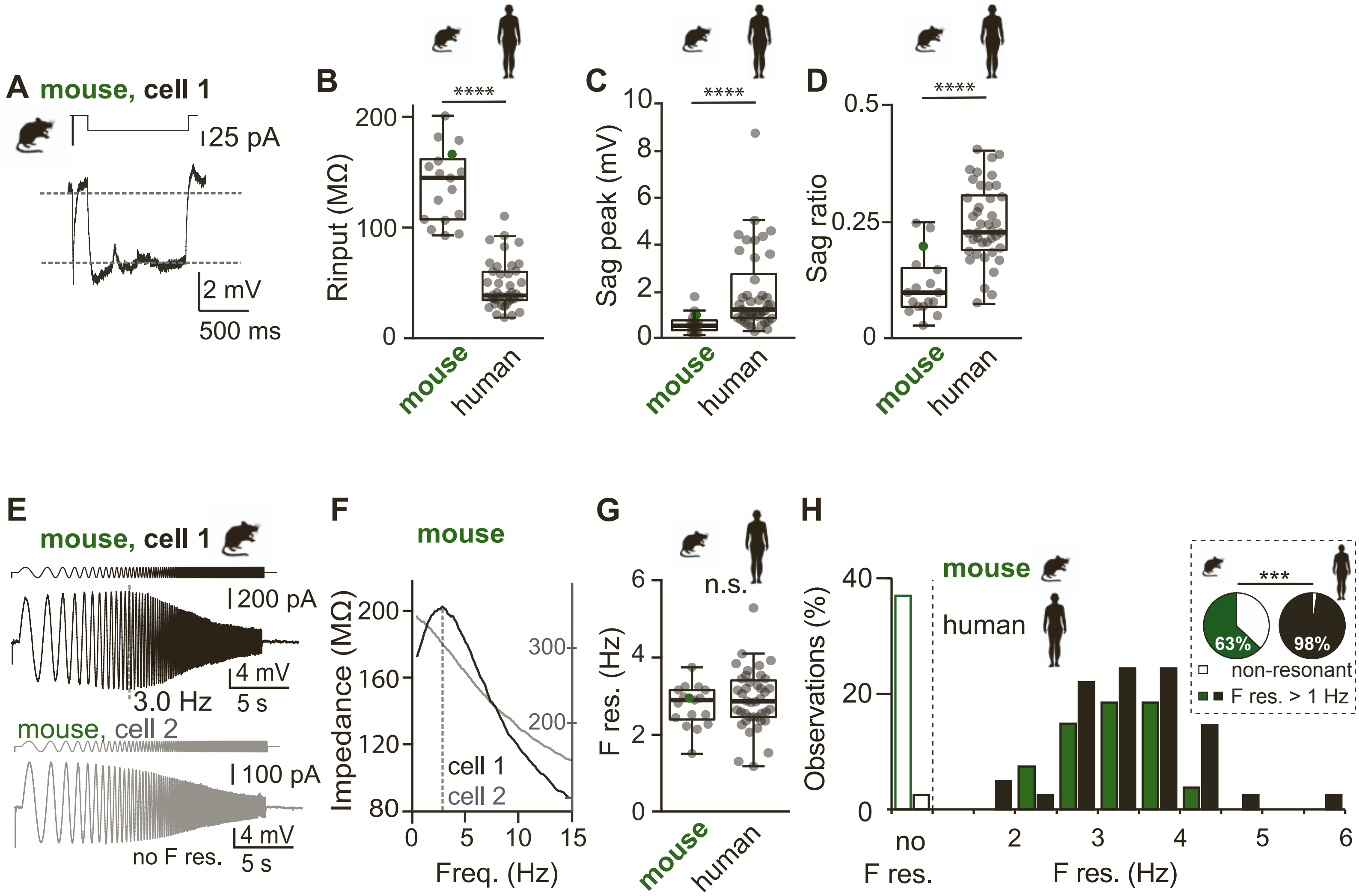

Fig.S2. Mouse CA1 neurons resonate at similar frequency, but the incidence is significantly lower. (A) Example mouse CA1 recording with hyperpolarization current to uncover Ih (or sag current). Dashed lines correspond to -70 mV and steady-state hyperpolarization (close to -73mV, see Methods), respectively. (B) Input resistance is significantly lower in human compared to mouse CA1 pyramidal neurons. (C, D) Maximal sag current amplitude (C) and sag ratio (D) is significantly higher in human compared to mouse CA1 pyramidal neurons. (E) Chirp protocol to extract resonance properties from mouse CA1 pyramidal neurons. Note two examples, of which cell 2 does not show resonant properties under control conditions. (F) Impedance profiles of example cells 1 and 2. Impedance of cell 2 is illustrated by 2nd y-axis. (G) Population statistics for preferred frequency (i.e. resonance frequency, n = 17, 2.9 Hz, 2.5 - 3.2 Hz, median, 1st – 3rd Quartile). (H) Histogram with distribution of resonance frequencies for human (black) and mouse (green), in addition to fraction of neurons without preferred frequency (open bars). Inset: pie charts representing the fraction of neurons with and without resonant properties. Note that in mouse, a large fraction of recordings did not show preferred resonance frequency (Human: 2.4% vs mouse: 37.0%, Fisher exact test, $p < 0.001$). Related to Figure 2.

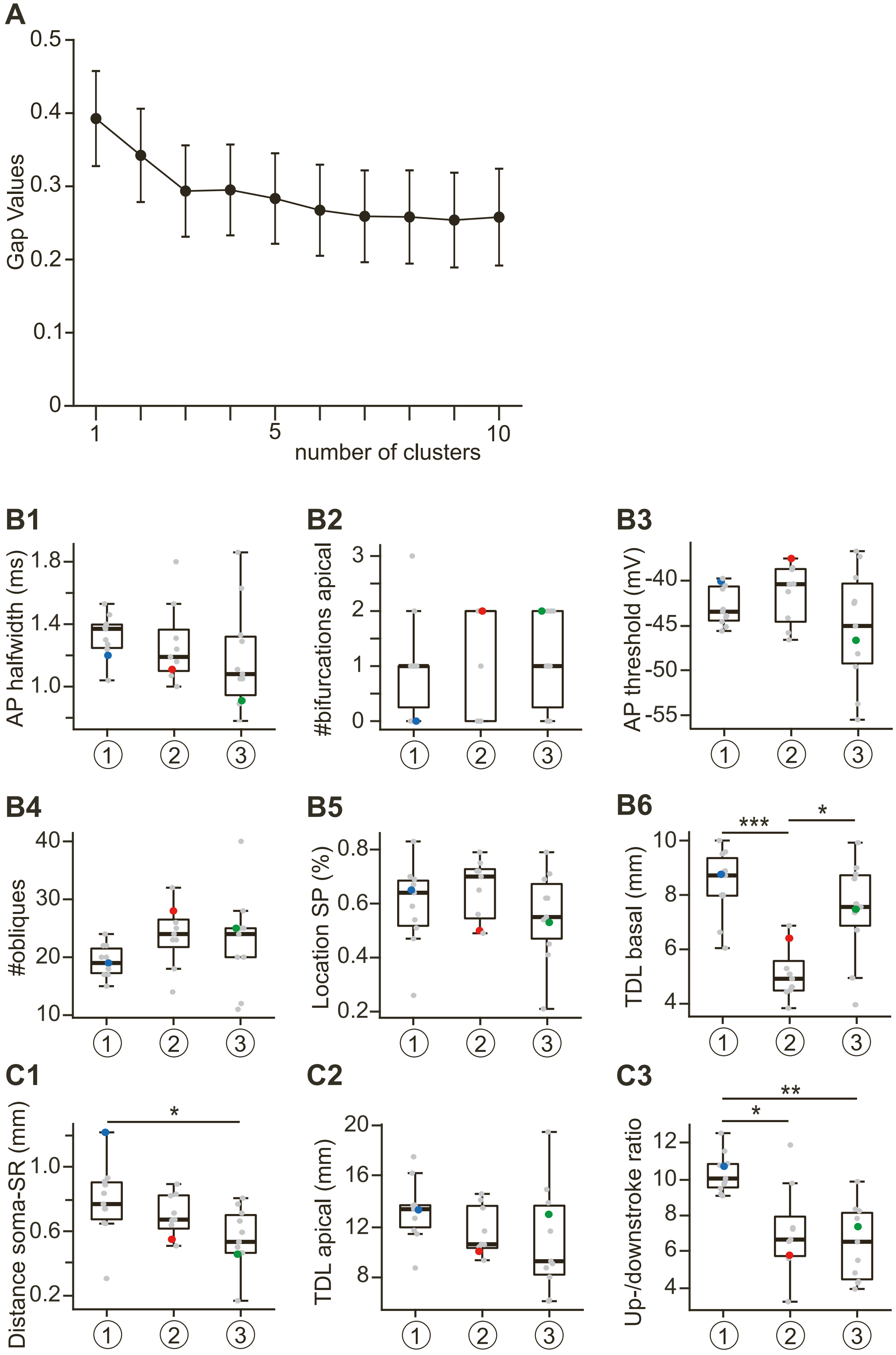

Fig.S3. Morpho-electric properties reveals cellular diversity along a multidimensional gradient. (A) Gap statistic values for estimating the number of clusters. Note that gap value is maximal for the condition of a single cluster. (B1-B6) Population statistics for the 6 additional principal components (for TDL, see main Fig.3). (C1-C3) Population statistics for three additional morpho-electric features. Note that significant differences between three dendrogram branches show a highly diverse pattern. Statistics: Kruskal-Wallis with Dunn's post-hoc test, * $p < 0.05$, ** $p < 0.01$, n.s.: not significant. Related to Figures 1, 2 and 3.

A

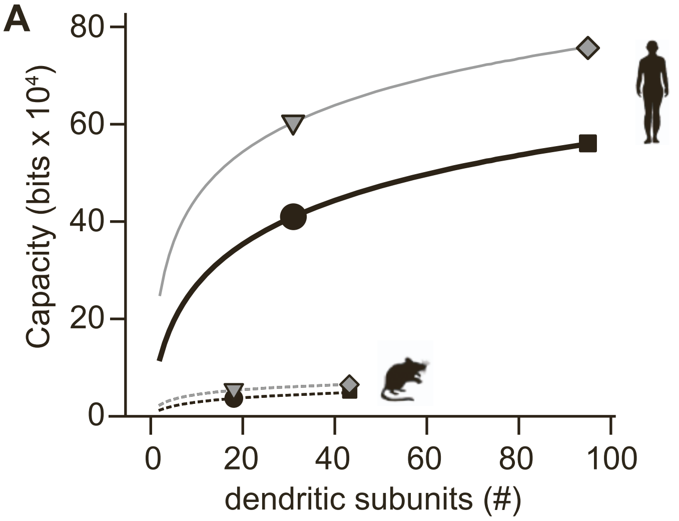

B

	m	d	memory capacity	memory capacity
●	trees	s / 5	41	4
■	terminal branches	s / 5	56	5
▽	trees	s / 1.2	60	6
◇	terminal branches	s / 1.2	76	7

Figure S4. : Structural complexity supports memory capacity. (A) Average memory capacity of human and mouse CA1 pyramidal neurons when considered as a two-layer model. Here, terminal branches are considered individual subunits (instead of trees, see main manuscript, Methods) and/or the number of synapses per connection is 1.2 (instead of 5, see main manuscript, Methods). (B) Parameter matrix to compute memory capacity. Related to Figure 4.

Table S1: Surgical cases.

Case #	Gender	Age (yrs)	Side	Diagnosis	Source	Assessment hippocampal anatomy
1	M	32	R	Epilepsy	VU Medical Center	w/o evident structural alteration
2	M	62		No information	Harborview Medical Center	No information
3	F	23	R	Epilepsy with tumor	VU Medical Center	w/o evident structural alteration
4	M	49	R	Epilepsy	VU Medical Center	w/o evident structural alteration
5	M	42	L	Epilepsy	VU Medical Center	w/o evident structural alteration
6	F	32	L	Epilepsy	VU Medical Center	w/o evident structural alteration

Table S2: Spine parameters to compute total synapse count. Related to Figure 4.

**Location**	**Density per 1 µm dendritic length**
**Basal tree**	2.43
**Apical trunk**	3.7
**Apical collaterals**	2.47
